# Supplementary material for: Lead-Free MDABCO-NH4I3 Perovskite Crystals Embedded in Electrospun Nanofibers
Source: Materials (Basel). 2022 Nov 25;15(23):8397. doi: 10.3390/ma15238397 (PMC9739599; doi:10.3390/ma15238397)
Supplement: Supplementary file 1 [file materials-15-08397-s001.zip › materials-1999692-supplementary.pdf]

Lead-free MDABCO-NH_4I_3 perovskite crystals embedded in electrospun nanofibers

Rosa Baptista [1,*], Gonçalo Moreira [1], Bruna Silva [1], João Oliveira [1], Bernardo Almeida [1], Cidália Castro [2], Pedro V. Rodrigues [2], Ana Machado [2], Michael Belsley [2] and Etelvina de Matos Gomes [2]

[a] Centre of Physics of Minho and Porto Universities (CF-UM-UP), University of Minho, Campus de Gualtar, 4710-057 Braga, Portugal

[b] Institute for Polymers and Composites, University of Minho, Campus de Azurém, 4800-058 Guimarães

[*]Corresponding author: rosa_batista@fisica.uminho.pt

Supplementary Information

SI1. XRD crystallites size

Estimates of the nanocrystal size, d, was obtained using the Scherrer equation

$$\beta_{hkl} = \frac{K\lambda}{d cos\theta} \qquad (1)$$

Here K is a correction factor to particle shape and λ the X-ray wavelength. In the absence of detailed shape information, a value of $K = 0.90$ is commonly used [1]. The full width of the diffraction peak at half the maximum (FWHM) gives the broadening of a (hkl) reflection and is expressed as β_{hkl}. To estimate the FWHM, first the main peaks were eliminated from the spectra and a cubic spline was globally fit to the resulting background using the MATLAB application "shape language modeling" [2].

Subsequently each individual background corrected peak was fit to and asymmetric Pseudo-Voight profile using the MATLAB nonlinear least squares fitting algorithm. The Pseudo-Voigt function is,

$$I = A\left[\eta L(x) + (1-\eta)G(x)\right] \qquad (2)$$

Representing a weighted linear superposition of Lorentz $L(x)$ and Gauss $G(x)$ functions. Here the parameter η represents the fractional contribution of the Lorentz function while the parameter A is proportional to the amplitude of the

diffraction peak above the background. The Lorentzian and Gaussian functions are:

$$L(x) = \frac{\left(2/\pi\beta^*\right)}{1+4x^2}$$
$$G(x) = \frac{2}{\beta^*}\sqrt{\frac{\ln(2)}{\pi}}\exp(-4\ln(2)x^2) \tag{2a}$$

written in terms of the scaled diffraction angle

$$x = \frac{2\left(2\theta - 2\theta_c\right)}{\beta^*} \tag{2b}$$

and the asymmetric width function [3]

$$\beta^* = \frac{2\beta}{1+\exp\left[a\left(2\theta-2\theta_c\right)\right]} \tag{2c}$$

In these expressions, the calculated peak position is $2\theta_c$ and a represents the asymmetry parameter.

The resulting fits are shown in Figure S1 a) to) while Tables S1 and S2 list the fit parameter values $\left(A,\eta,\theta_c,\beta,a\right)$ for the two peaks $(11\bar{1})$ and (200). The red shading in Figure S1 represents the 68% confidence intervals of the fit peaks.

Table S1: Fit parameter values for the MDABCO-NH_4I_3 embedded into PVC, PA66 and PMMA correspondent to Bragg reflection $(11\bar{1})$.

	A	η	$2\theta_c$	β	a
MDABCO-NH_4I_3@ PVC	9.02	1.00	21.947º	0.124º	-6.35
MDABCO-NH_4I_3@ PA66	12.639	1.00	21.941º	0.099º	-2.45
MDABCO-NH_4I_3@ PMMA	19.50	1.00	21.883	0.100º	4.846

Table S2: Fit parameter values for the MDABCO-NH_4I_3 embedded into PVC, PA66 and PMMA correspondent to Bragg reflection (200).

	A	η	$2\theta_c$	β	a
MDABCO-NH_4I_3@ PVC	12.84	0.60	24.676º	0.158º	16.51
MDABCO-NH_4I_3@ PA66	9.07	1.00	24.702º	0.103º	6.37
MDABCO-NH_4I_3@ PMMA	14.10	0.65	24.649º	0.121º	3.53

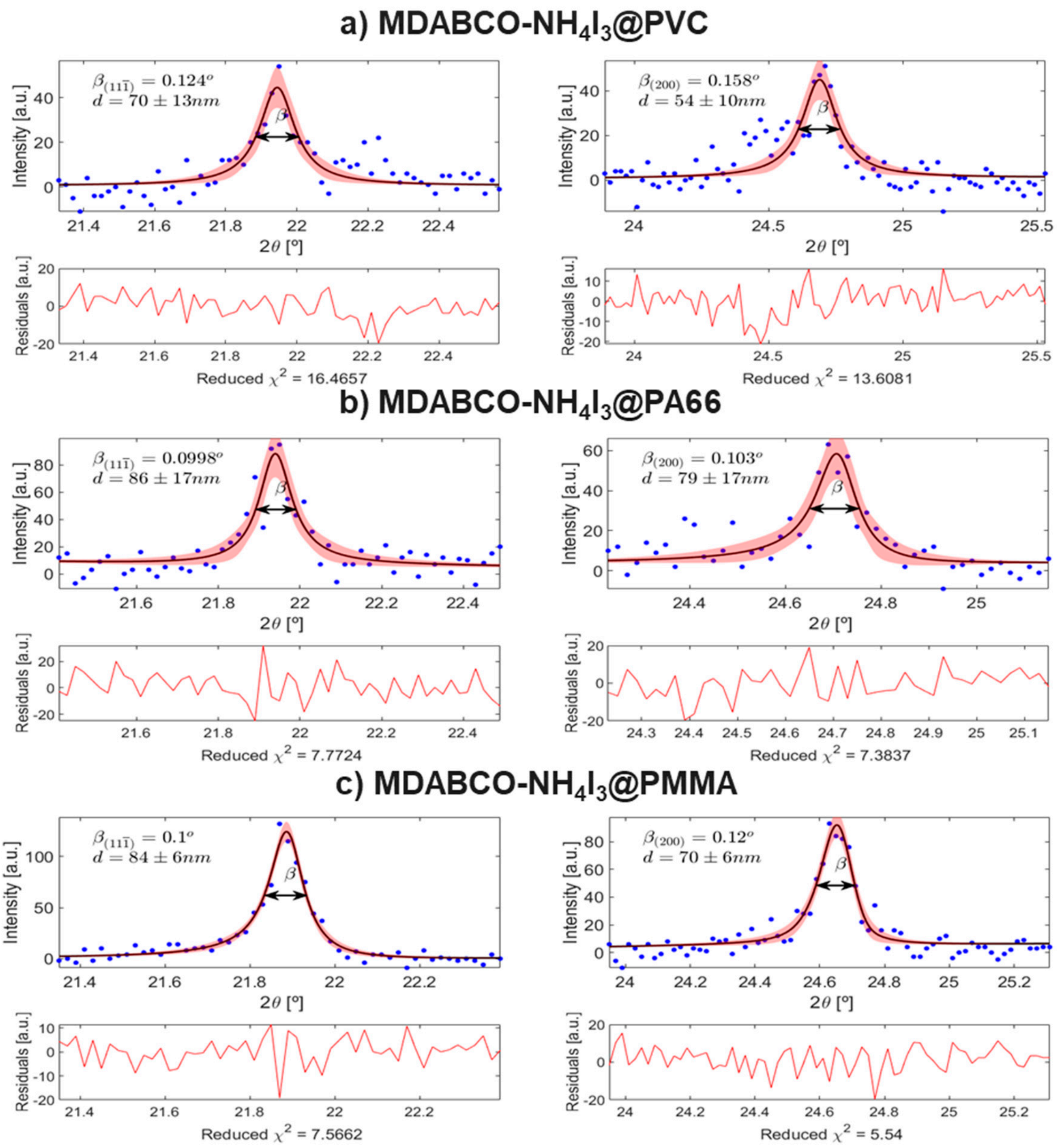

Figure S1. Asymmetric Pseudo-Voigt fits of the $(11\bar{1})$ and (200) Bragg reflections for MDABCO-NH_4I_3 crystals inside a) PVC, b) PA66 and c) PMMA nanofibers. The width of the reflections at half the maximum β and the nanocrystals average size d is indicated as well the refit residuals.

S2. UV-Visible Reflectance Spectra

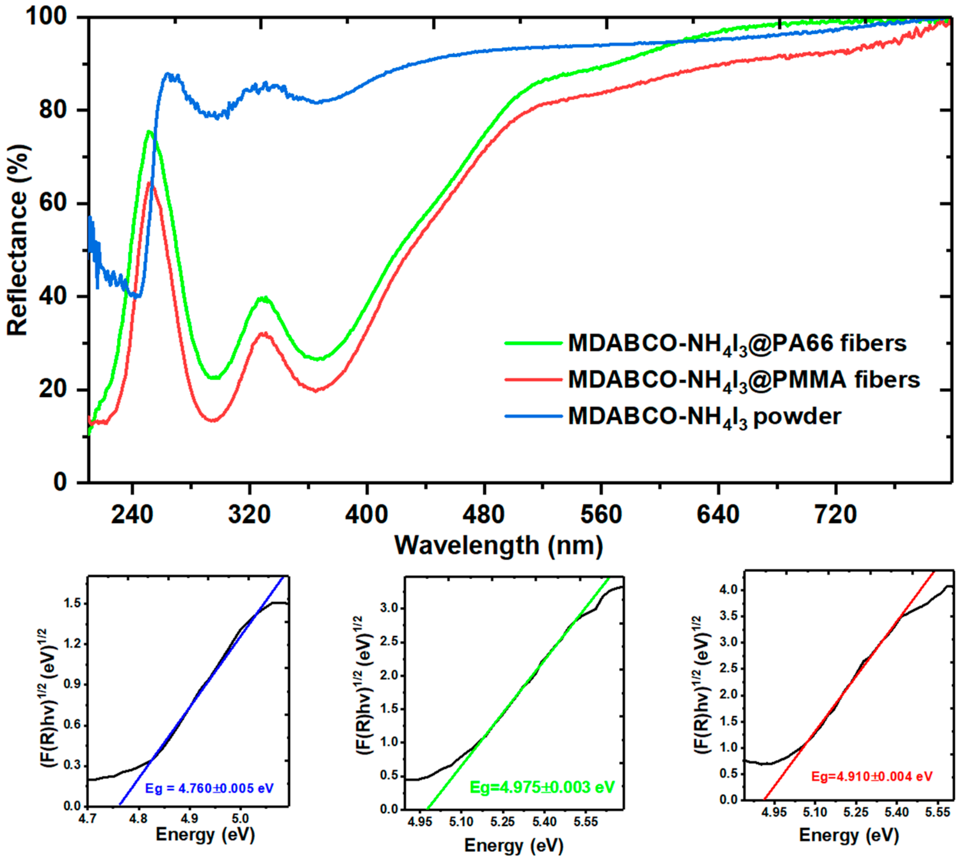

Figure S2. UV–vis reflectance of MDABCO-NH_4I_3 powder pellet and electrospun fibers. The inset shows the Kubelka-Munk function indicating a band gap energy of 4.760 eV for the polycrystalline perovskite, 4.975 eV and 4.910 eV for MDABCO-NH_4I_3@PA66 and MDABCO-NH_4I_3@PMMA nanofibers, respectively.

S3. DSC analysis

In figure S3, the measured DSC of synthesized MDABCO-NH_4I_3 perovskite crystals, showed the ferroelectric-paraelectric phase transition to occur at 443 K with area 19.9 J/g. MDABCO-NH_4I_3 perovskite crystals start to decompose above 460 K with the formation of NH_4IO_3. Cooling down from 473 K, shows a peak at 350 K, area -13.26 J/g. During the second heating, a peak with maximum at 358 K and area 12.3 J/g is observed. These two peaks correspond respectively to the paraelectric to ferroelectric and ferroelectric-paraelectric phase transitions of

NH_4IO_3 compound, which was formed after the first heating [4]. The perovskite phase transition at 443 K is not recovered, which reveals that crystal thermal degradation starts above 460 K[5].

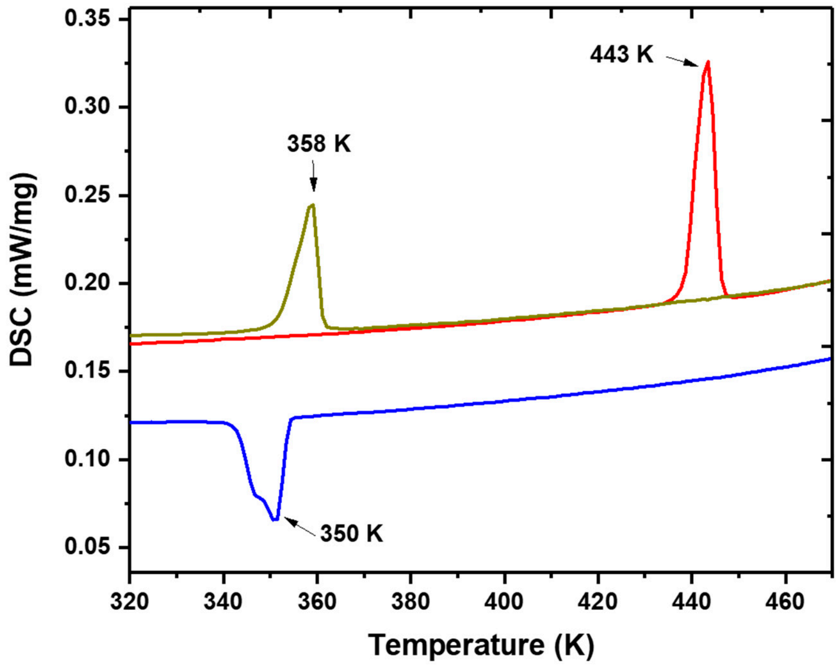

Figure S3. DSC spectra of synthesized MDABCO-NH_4I_3 perovskite crystals.

S4. RAMAN Spectra

In Figure S4, the measured Raman modes between $50 - 600\ cm^{-1}$ are shown for a MDABCO-NH_4I_3 polycrystalline sample, for electrospun fibers of PVC and MDABCO-NH_4I_3@PVC. In the spectra, the low frequency modes are due to lattice vibrations. From the two bands at $68\ cm^{-1}$ and $103\ cm^{-1}$ present in the polycrystalline sample, only the second band (now shifted to $109\ cm^{-1}$) remains intense for the perovskite nanocrystals embedded in the polymer fibers. The strong decreasing in intensity of the $68\ cm^{-1}$ band is probably the result of constrains imposed by the polymer matrix on the lattice vibrations modes, due to crystal confinement.

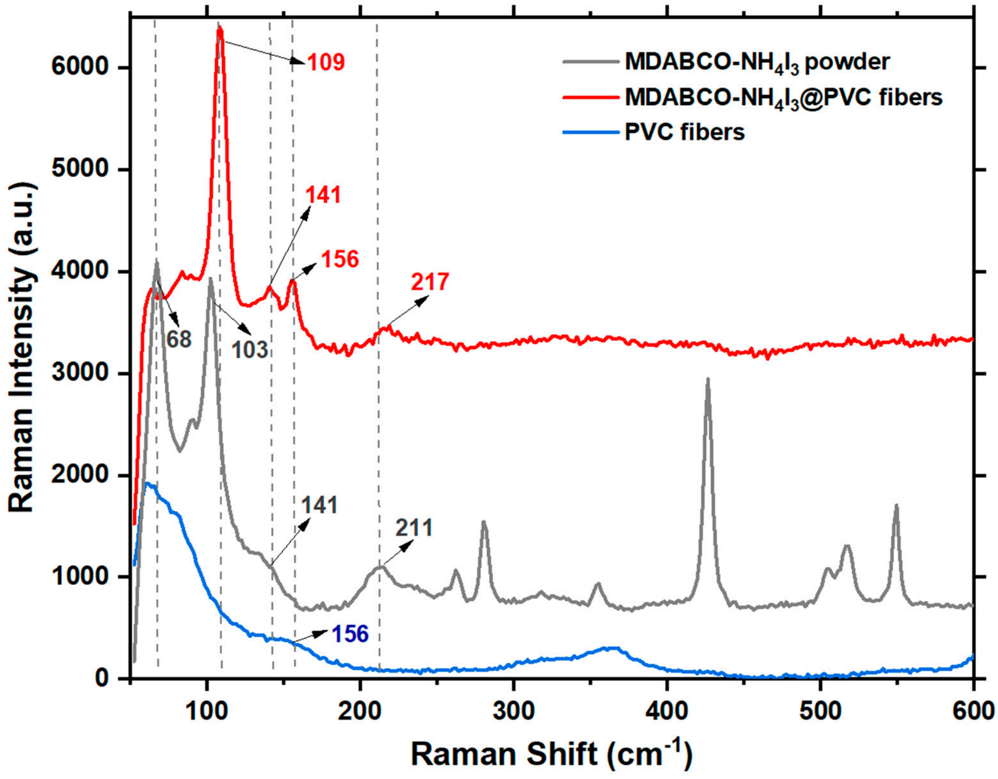

Figure S4. Raman spectra for MDABCO-NH_4I_3 polycrystalline sample (black line), for electrospun fibers of PVC (blue line) and MDABCO-NH_4I_3@PVC fibers (red line), expansion between $50 - 600\ cm^{-1}$.

The weak broad bands centred at $141\ cm^{-1}$ for polycrystalline MDABCO-NH_4I_3 and $156\ cm^{-1}$ for PVC fibers, are resolved into well-defined bands for MDABCO-NH_4I_3@PVC fibers. The weak band for MDABCO-NH_4I_3 at $211\ cm^{-1}$ is slightly shifted to $217\ cm^{-1}$, for the perovskite incorporated into the fibers.

Noticed that for MDABCO-NH_4I_3@PVC fibers, none of the Raman peaks between $250 - 600\ cm^{-1}$ are perceptible, while they are present in the MDABCO-NH_4I_3 perovskite spectra. We may conclude that the polymer matrix strongly constrains these perovskite vibrational modes as mentioned above, which is expectable as the fibers were electrospun from a precursor solution with ration of MDABCO-NH_4I_3 to PVC of 1:5.

S5. FTIR-ATR analysis

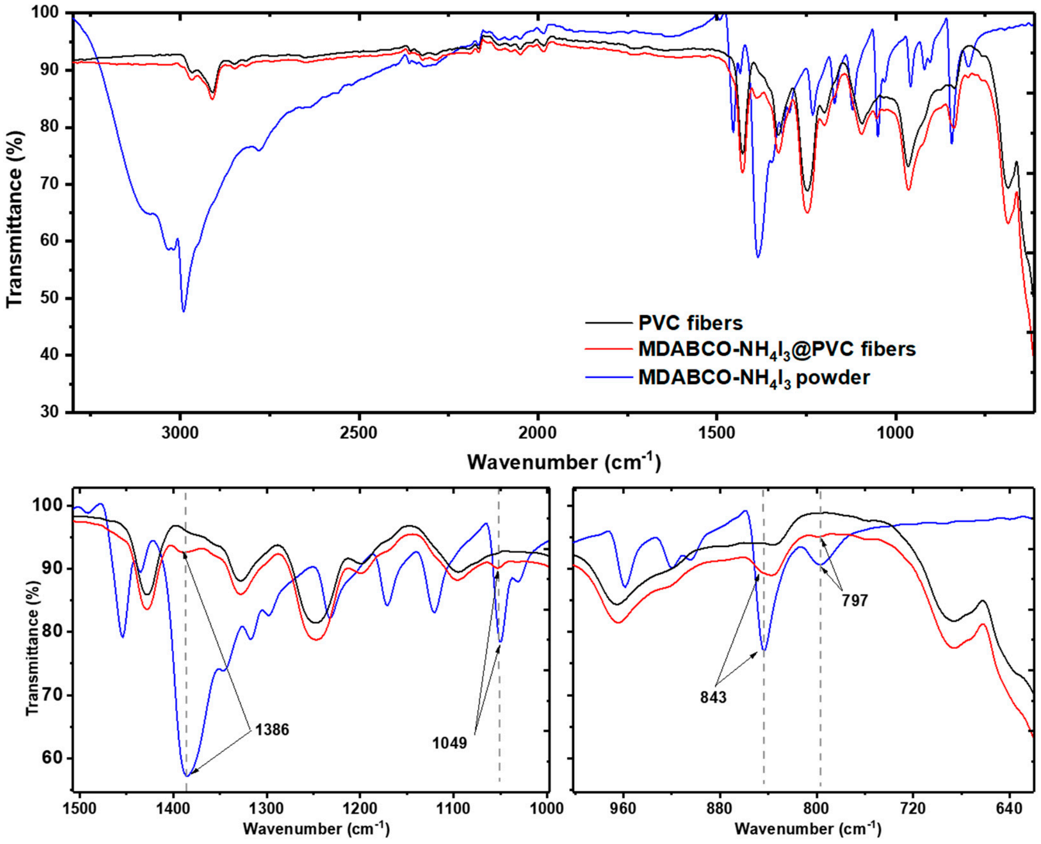

Figure S5. FTIR-ATR spectra of PVC polymer, MDABCO-NH_4I_3 powder and MDABCO-NH_4I_3@PVC fibers, a) full measurement and b) expansion between $1000 - 1500\ cm^{-1}$ and $600 - 1000\ cm^{-1}$.

In Figure S5 b), the measured FTIR-ATR between $1500 - 600\ cm^{-1}$ is shown for a MDABCO-NH_4I_3 polycrystalline sample, for electrospun fibers of PVC and MDABCO-NH_4I_3@PVC. Slight differences were observed between the spectra of electrospun fibers of PVC and MDABCO-NH_4I_3@PVC, and just few bands (the most intense) were possible to be assigned to MDABCO-NH_4I_3 perovskite nanocrystals at the $1386\ cm^{-1}$, $1049\ cm^{-1}$, $843\ cm^{-1}$ and $797\ cm^{-1}$. However, the strong decreasing in intensity observed for the perovskite bands on electrospun fibers of MDABCO-NH_4I_3@PVC sample is due to the sobreposition of the polymer bands to those of MDABCO-NH_4I_3 crystals. As already mentioned, the ratio perovskite and polymer are 1:5, on the precursor solution used to fabricate

the electrospun fibers. Therefore, is expected that the only the stronger perovskite bands will be observed. That contained a concentration constrain imposed by the polymer matrix on the IR bands.

S6. Piezoelectric nanogenerator

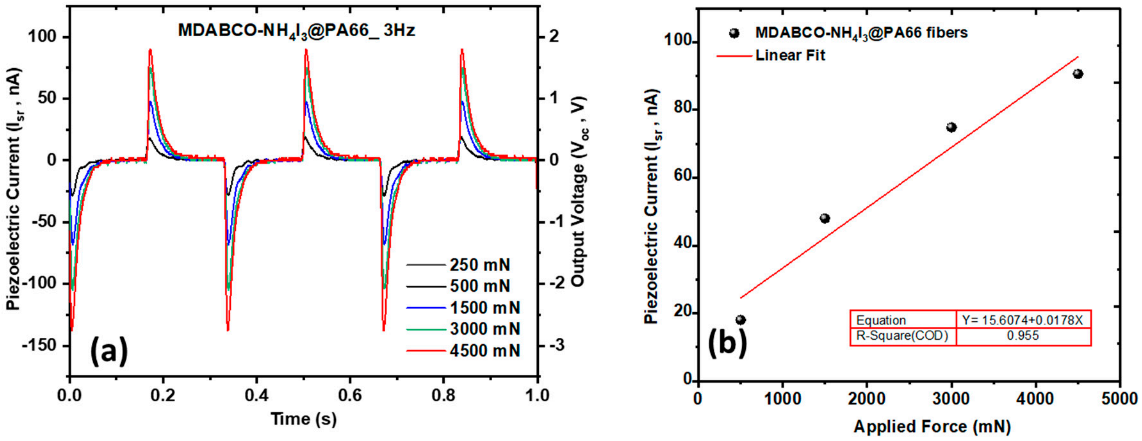

Figure S6. MDABCO-NH4I3@PA66 fibers (a) Piezoelectric current as function of time, for several applied forces and (b) linear fitting.

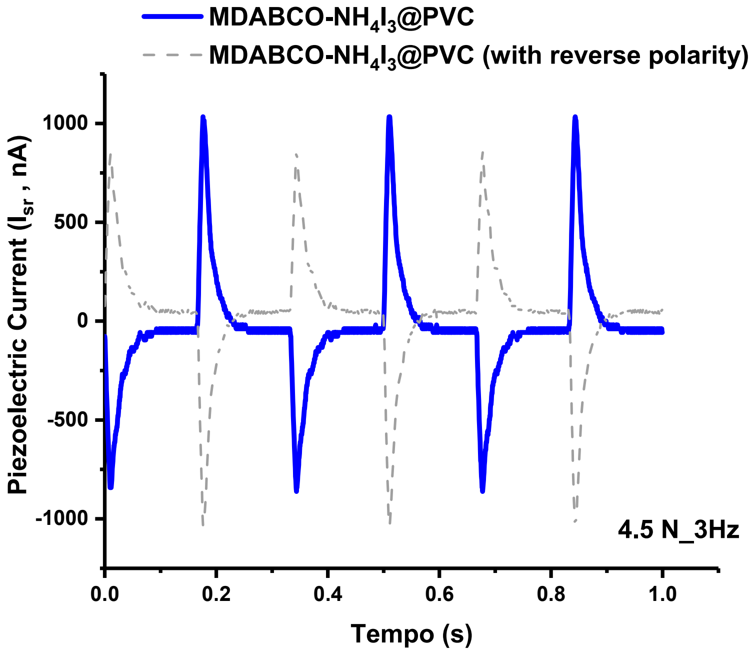

Figure S7. Output voltage generated from MDABCO-NH4I3@PVC nanofiber mat with reverse polarity.

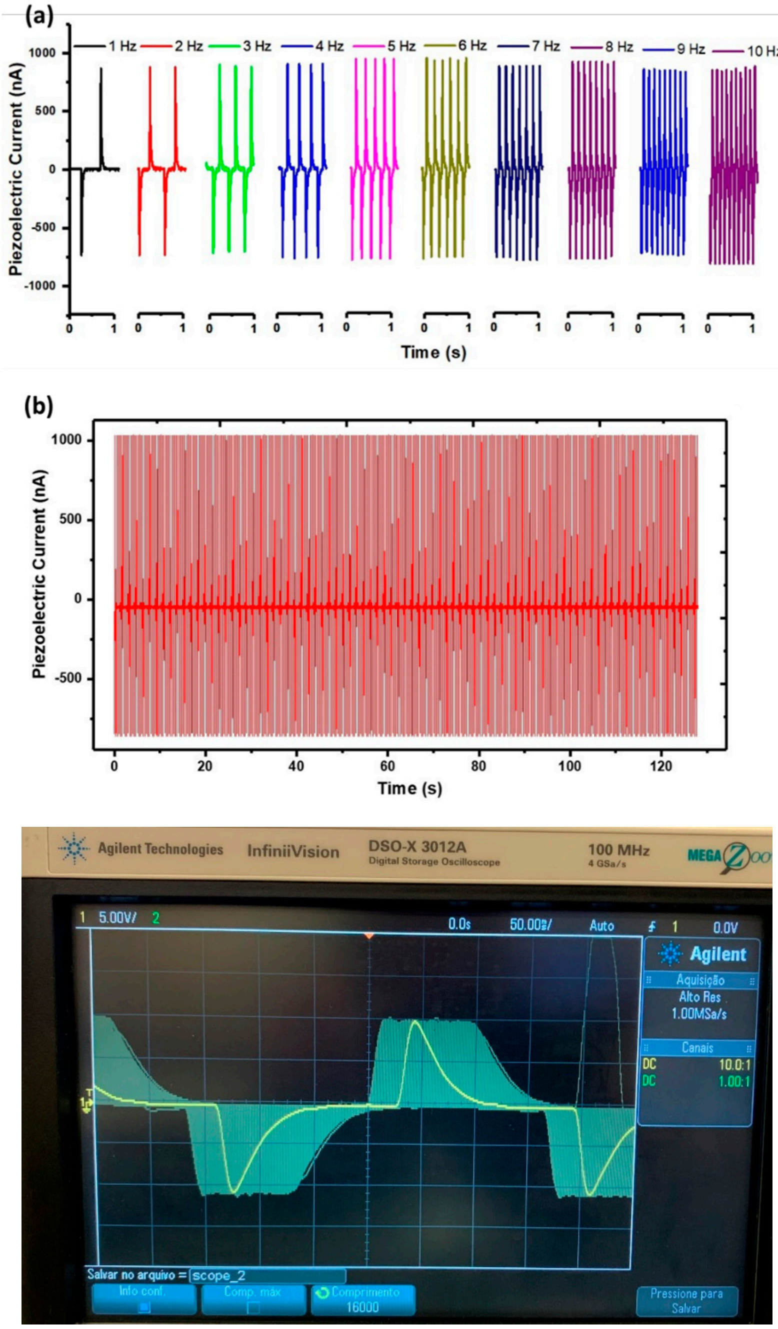

Figure S8. MDABCO-NH_4I_3@PVC nanofiber mat piezoelectric current generated a) for frequencies between 1Hz and 10 Hz and b) a 130 s interval under a periodical force applied with 4.5 N. A stability test was performed during a time interval of 4 h under a periodical force applied with 2.7 N at a frequency of 3 Hz (oscilloscope image).

S9. Mechanical Properties

Tensile strain applied to PVC and MDABCO-NH_4I_3@PVC electrospun fiber mat are shown in Figure S9. The results from tensile measurements show that MDABCO-NH_4I_3@PVC fibers exhibit good mechanical performance with an average increase of 14% on tensile strength (reaching ~ 4.0 MPa) and an increase of 40% on the strength at yield reaching (~ 2.5 MPa) when compared with PVC fibers. More importantly, the Young modulus increases from ~20 MPa in PVC fibers to ~58 MPa in MDABCO-NH_4I_3@PVC fibers, an increase of around 66%. This significant increase of the Young modulus of perovskite doped electrospun fibers indicates that composite MDABCO-NH_4I_3@PVC are mechanically stronger than neat polymer fibers. This is very important for the performance of MDABCO-NH_4I_3@PVC fiber mat as nanogenerator as it will increase its capability of supporting longer times under applied external force.

The decrease of 30% on the strain at break for doped fibers indicates a decrease in their plasticity, which might result from the presence of nanocrystals inside the PVC polymer which inhibits the capability for the PVC chains to flow between.[6]

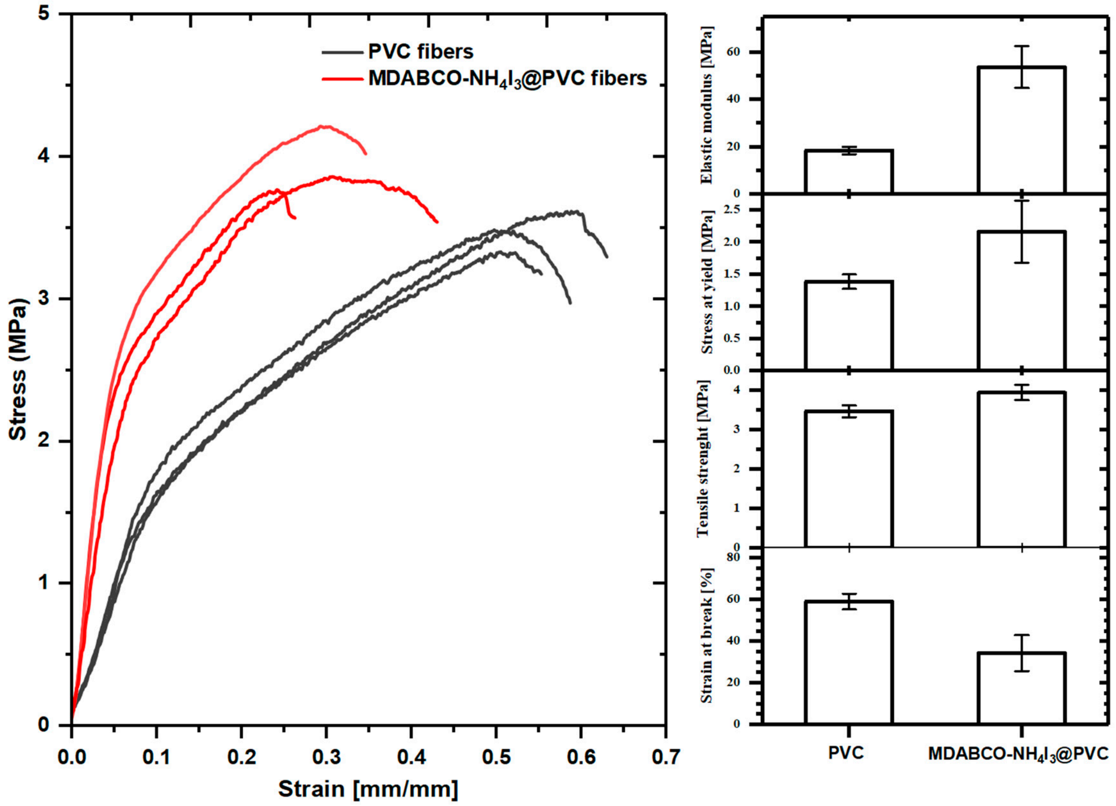

Figure S9. Elastic modulus, stress at yield, tensile strength and strain at break of PVC and MDABCO-NH_4I_3@PVC electrospun fibers.

References

(1) Klug, H.; Alexander, L., *X-Ray Diffraction Procedures 2nd edn*, Ch. 9. Wiley: 1974.

(2) John D'Errico (2022). SLM - Shape Language Modeling. https://www.mathworks.com/matlabcentral/fileexchange/24443-slm-shape-language-modeling), MATLAB Central File Exchange. Retrieved July 5, 2022.

(3) Stancik, A. L.; Brauns, E. B. A simple asymmetric lineshape for fitting infrared absorption spectra. *Vib Spectrosc* **2008,** *47* (1), 66-69, DOI: https://doi.org/10.1016/j.vibspec.2008.02.009.

(4) Keve, E. T.; Abrahams, S. C.; Bernstein, J. L. Pyroelectric Ammonium Iodate, a Potential Ferroelastic: Crystal Structure. *The Journal of Chemical Physics* **1971,** *54* (6), 2556-2563, https://doi.org/10.1063/1.1675212.

(5) Sirbu, D.; Tsui, H. C. L.; Alsaif, N.; Iglesias-Porras, S.; Zhang, Y.; Wang, M.; Liu, M.; Peacock, A. C.; Docampo, P.; Healy, N. Wide-Band-Gap Metal-Free Perovskite for Third-Order Nonlinear Optics. *Acs Photonics* **2021,** *8* (8), 2450-2458, https://doi.org/10.1021/acsphotonics.1c00687.

(6) Lee, D. W.; De Los Santos V, L.; Seo, J. W.; Felix, L. L.; Bustamante D, A.; Cole, J. M.; Barnes, C. H. W. The Structure of Graphite Oxide: Investigation of Its Surface Chemical Groups. *The Journal of Physical Chemistry B* **2010,** *114* (17), 5723-5728, https://doi.org/10.1021/jp1002275.
